# Cardiometabolic outcome of MyBFF@school intervention program among secondary schoolchildren: a cluster randomized controlled trial

**DOI:** 10.1186/s12889-026-26289-5

**Published:** 2026-02-27

**Authors:** Muhammad Yazid Jalaludin, Farah Aqilah Roslan, Fazliana Mansor, Fuziah Md. Zain, Janet Yeow Hua Hong, Ruziana Mona Wan Mohd Zin, Nur Zati Iwani Ahmad Kamil, Abqariyah Yahya, Zahari Ishak, Rusidah Selamat, Abdul Halim Mokhtar

**Affiliations:** 1 Department of Pediatrics, Faculty of Medicine, University of Malaya, University of Malaya, Wilayah Persekutuan Kuala Lumpur, 59100 Kuala Lumpur, Malaysia; 2 Endocrine and Metabolic Unit, Institute for Medical Research, National Institute of Health (NIH), Ministry of Health Malaysia, Setia Alam, 40170 Shah Alam, Selangor Malaysia; 3 Department of Pediatrics, Putrajaya Hospital, Ministry of Health Malaysia, Jalan P9, Pusat Pentadbiran Kerajaan Persekutuan Presint 7, 62250 Putrajaya, Wilayah Persekutuan Putrajaya Malaysia; 4 Department of Social and Preventive Medicine, Faculty of Medicine, University of Malaya, Wilayah Persekutuan Kuala Lumpur, 50603 Kuala Lumpur, Malaysia; 5 Wellbeing Research Center, Faculty of Social Sciences and Liberal Arts, UCSI University, 56000 Kuala Lumpur, Malaysia; 6 Nutrition Division, Federal Government Administrative Centre, Ministry of Health Malaysia, Level 1, Block E3, Complex E, 62590 Putrajaya, Wilayah Persekutuan Putrajaya Malaysia; 7 Sports Medicine Unit, Faculty of Medicine, University of Malaya, 59100 Kuala Lumpur, Wilayah Persekutuan Kuala Lumpur Malaysia; 8 Centre for Sports and Exercise Science, University of Malaya, Kuala Lumpur, Malaysia

**Keywords:** Diabetes, Insulin resistance, Blood pressure, Lipid profile, School-based intervention, Adolescent obesity

## Abstract

**Background:**

Type 2 diabetes, hypertension, and dyslipidemia are comorbidities associated with obesity in children and adolescents. This school-based, cluster-randomized controlled study evaluated the effectiveness of a 6-month intervention (MyBFF@school) on cardiometabolic markers among overweight and obese Malaysian adolescents.

**Methods:**

This study involved proportionate stratified random sampling of government schools in three selected states in Malaysia. Fifteen out of 416 schools were selected. Schoolchildren aged 13–16 years with body mass index (BMI) z-score greater than + 1 SD (WHO) were recruited. The outcome measures included fasting plasma glucose, fasting insulin, homeostatic model assessment of insulin resistance (HOMA-IR), lipid profiles, TG:HDL-C ratio and blood pressure.. Repeated measures ANOVA and ANCOVA were used to assess within- and between-group effects.

**Results:**

From 1041 adolescents, 552 (intervention = 306 and control = 246) had complete fasting bloods taken at baseline and month˗6. BMI z-scores decreased significantly within both groups, but no between-group differences were observed. Notably, BMI increased (albeit small) within the intervention group compared with baseline (mean difference (MD) 0.17 kg/m^2^; 95% confident interval (CI) 0.02, 0.32)*.* A greater increase of HDL-C was observed in the intervention group at month-6 (MD = 0.06 mmol/L, 95% CI0.10,0.20). Fasting insulin was significantly decreased within the intervention group (MD = − 2.24 μU/mL (95% CI − 3.37, − 1.17) but no significant changes were detected between groups. Unexpectedly, fasting plasma glucose showed significant increase in both groups post-intervention (MD = 0.38, 95%CI 0.28,0.48). Similarly, although within normal range, significant increase of total cholesterol, TG and LDL-C, (MD = 0.36, 95%CI 0.25,0.47, MD = 0.26 95%CI 0.17,0.35 and MD = 0.30 95%CI 0.18,0.42 respectively) was observed. Additionally, significant increase of systolic and diastolic blood pressure was observed in the intervention group post- intervention compared to control group (MD = 3.68 95%CI 2.01,5.35 and MD = 4.29 95%CI 2.85,5.73) respectively.

**Conclusion:**

The MyBFF@school programme, resulted in reductions in BMI z-score in both groups; however, increases in BMI and several cardiometabolic markers were also observed within the intervention group. These mixed findings underscore the importance of evaluating both anthropometric and metabolic outcomes when assessing the effectiveness and safety of obesity interventions in adolescents.

**Trial registration:**

Clinical trial number: NCT04155255, November 7, 2019 (Retrospective registered). National Medical Research Register: NMRR-13–439-16563. Registered July 23, 2013. The intervention program was approved by the Medical Research and Ethics Committee (MREC), Ministry of Health Malaysia and Educational Planning and Research Division (EPRD), Ministry of Education Malaysia. It was funded by the Ministry of Health Malaysia.

## Background

The worldwide prevalence of overweight and obese children and adolescents has increased drastically in recent decades [1]. In Malaysia, few studies have reported the prevalence of overweight and obese secondary school students aged 13 to 15 years, which ranges from 8.2% to 19.5% [2–4]. Clinically, overweight and obese adolescents may suffer from short-term consequences such as asthma, systemic inflammation, and cardiovascular risk, together with long-term consequences such as diabetes, hypertension, stroke, certain types of cancer, and premature death [5, 6].

Type 2 diabetes mellitus (T2DM) is characterized by insulin resistance and an ongoing loss of endogenous insulin secretion [7]. Previous literature indicates that the incidence of T2DM in youth has increased dramatically over the last few decades [8]. Children and adolescents with T2DM or insulin resistance are expected to have a higher risk of developing microvascular and macrovascular complications and thus mortality in early adulthood because of longer durations of the disease and greater durations of glycemic exposure [9, 10]. The triglycerides to high-density lipoprotein cholesterol (TG:HDL-C) ratio has been reported to be closely linked to insulin resistance [11]. However, earlier studies have shown conflicting findings in proving an association between TG:HDL-C ratio and insulin resistance [12–15]. Despite this, the usefulness of simple and inexpensive diagnostic tools may help in the prevention and detection of T2DM [16].

Dyslipidemia patterns associated with childhood obesity consist of a combination of elevated total cholesterol, triglycerides, low-density lipoprotein cholesterol (LDL-C), and decreased high-density lipoprotein cholesterol (HDL-C) [17]. In addition, insulin resistance is a common feature in obese children and adolescents and contributes significantly to the development of the combined dyslipidemia of obesity by enhancing hepatic delivery of non-esterified free fatty acids for triglyceride production and sequestration into triglyceride-rich lipoproteins [18]. A previous study by Elmaogullari et al. [19] showed that 42.9% of children aged 2 to 18 years were diagnosed with dyslipidemia and increased age and/or BMI were associated with an increased prevalence of dyslipidemia.

Hypertension is the most common chronic condition found in overweight and obese adolescents and can lead to increased risk of adulthood mortality [20]. This was evidenced by a large cohort study involving children and adolescents, which showed higher blood pressure at baseline for those with obesity and severe obesity and greater risk of developing hypertension in adulthood than those with lower body mass index (BMI) [21]. A recent study by Liew et al. [22] reported that the prevalence of hypertension among Malaysian adolescents aged 13 to 17 years was 24.5% and recorded a strong association of hypertension with BMI (OR = 4.05, 95% CI 1.67, 9.79).

Childhood and adolescence are crucial stages in the development of a healthy lifestyle, since most habits acquired during this period of life are sustained into adulthood [23]. Educational and social sectors, especially schools, are ideal spaces for the development of health-oriented interventions, since much adolescence is spent in school. A number of studies, for example, a school-based intervention in various countries, have shown that different types of physical activity practices reduce cardiometabolic risk factors [24, 25] and indicate that such interventions should focus both on promoting physical activity and nutrition education [23].

In Malaysia, data on effective intervention programs focusing on adolescent obesity are still lacking. To the best of our knowledge, My Body is Fit and Fabulous at School (MyBFF@school) initiative is the first large-scale school-based intervention study involving overweight and obese schoolchildren aged 13, 14, and 16 years in Malaysia. The objective of this study is to evaluate the effectiveness of school intervention on the cardiometabolic markers such as blood glucose, insulin resistance, lipid profiles, and blood pressure after a 6-month intervention period.

## Methods

### Study design and participants

MyBFF@school initiative is a multi-component intervention study comprising physical activity, nutrition, and psychology aspects. This study was a school-based cluster randomized control trial involving overweight and obese schoolchildren and adolescents. A proportionate stratified random sample from all types of government schools in the states of Selangor, Negeri Sembilan, and Federal Territory of Kuala Lumpur, Malaysia, was used in the sampling. The intervention period began in February 2016 and ended in August 2016.

MyBFF@school program incorporated physical activity in the form of small-sided games (SSG), nutrition, and psychology components. SSG (football, handball, and fun games) sessions were conducted for 40 min as allocated in the school curriculum. Two sessions were organized per week; therefore, a total of 80 min per week was spent on SSG activities. Nutrition education intervention (NEI) utilized the nutrition education modules (NEM) adapted from the Malaysian Childhood Obesity Treatment Trial (MASCOT)'s modules. This intervention consists of classroom lectures, hands on practical sessions and interactive activities, and were carried out to strengthen nutrition knowledge, attitude, and practices. The main themes in the psychology modules were self-esteem, friendship, assertiveness, and positive thinking for a healthy lifestyle and stress management, guided by trained research assistants. Motivational talks and interactive activities were the two main mechanisms used to achieve the objectives, followed by a reflection session at the end of the activities. Nutrition and psychology sessions were conducted by trained research assistant at alternate week during the period of co-curriculum activities with 45–60 min per session.

Full-time trained personnel (research assistant) were assigned to conduct the modules for each component. They were stationed at each school to ensure delivery of the intervention package and the commitment of the schoolchildren to this study. On top of this, the research team monitored the schoolchildren on a monthly basis. Formal assessment was performed at baseline, three and six months.

**Fig. 1:**
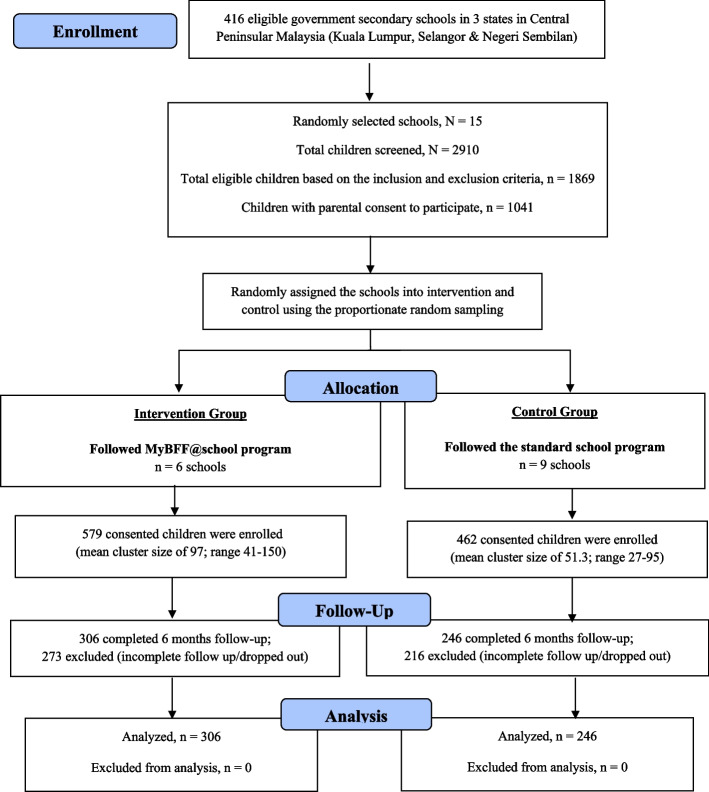
CONSORT diagram for clinical component in MyBFF@school

**Fig. 2:**
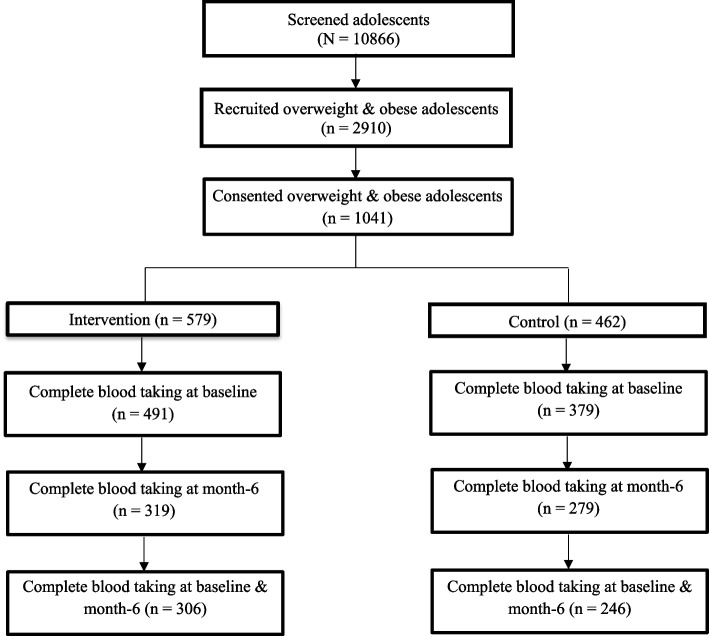
Flowchart of the MyBFF@school study

The design of the study and its implementation are described in detail by Mokhtar et al. [26]. A total of 2910 secondary schoolchildren aged 13,14, and 16 years were screened, of which 1869 overweight and obese children were invited to join the study. 1041 children consented to their participation in the study, of whom 579 were placed in the intervention group and 462 were placed in the control group (Fig. 1). Complete (taken at baseline and at six month) blood samples were available from 552 (53.0%) students (intervention = 306 and control = 246), and analyses were made on students from both the intervention and control groups at baseline and after six months (Fig. 2).

### Demographic and anthropometric measurements

All students in the participating schools were screened, and those who fulfilled the inclusion and exclusion criteria were invited to participate in this study. The inclusion criteria were secondary schoolchildren aged 13–16 years old, overweight or obese, with a BMI z-score of more than + 1 SD based on the WHO 2007 Growth Reference. The exclusion criteria were schoolchildren with BMI z-score below and/or equal to + 1 SD, with physical or mental disability, medical conditions that prevented their participation in moderate to vigorous physical activities, co-morbidities that may interfere with the study (such as T2D, hypertension, nephritic syndrome, epilepsy, congenital heart disease and skeletal anomalies), or a requirement for steroids, anti-epileptic treatments, or methylphenidate. Only those who consented were allowed to participate in this study.

Demographic data such as age, gender, ethnicity, and school locality were obtained by medical officers and recorded in a booklet for each student. BMI calculations were performed by dividing weight in kilograms by the square of height in meters (kg/m^2^) [27], and results were categorized as overweight, obese, or morbidly obese based on World Health Organization (WHO) BMI z-score criteria [27, 28]. WHO AnthroPlus 2007 software was used to calculate the distribution of BMI z-scores with indications corresponding to overweight (> + 1.0 SD), obese (> + 2.0 SD), and morbidly obese (> + 3.0 SD) [27].

### Clinical measurements

Blood pressure was measured manually by trained staff by using a mercury sphygmomanometer (Accoson, UK) with an appropriate cuff size for each individual. Measurements were obtained with students in a seated position, with the right upper arm positioned at the heart level and feet flat on the ground. Two measurements were taken with a 5 min interval for improved accuracy, and the mean value was recorded [28].

Venous blood samples were taken by trained nurses or medical officers after a minimum of 8 h of fasting. Two milliliters (mL) of blood were collected in sodium fluoride tubes for glucose measurements and another 5 mL in plain test tubes in order to measure insulin levels and lipid profiles (total cholesterol, triglycerides, HDL-C, and LDL-C). All blood samples were labeled and transported in an icebox to the central laboratory at the Institute for Medical Research within 2 h of collection. Serum total cholesterol, triglycerides, HDL-C, LDL-C, and plasma glucose were analyzed by Randox Laboratories (Antrim, UK) using an auto chemical analyzer (Dirui CS-400, China). Fasting insulin levels were measured using an automated enzyme immunoassay analyzer (TOSOH AIA-360, Japan).

### Definition of measures

Diabetes is determined according to fasting plasma glucose, which is divided into normal (< 5.6 mmol/L), pre-diabetes (5.6–7.0 mmol/L), and diabetes (≥ 7.0 mmol/L) levels [28]. Homeostatic model assessment of insulin resistance (HOMA-IR) is calculated by multiplying the value of fasting insulin with fasting plasma glucose and dividing this value by 22.5 [29]. Values may be characterized as insulin sensitive (< 4.0) and insulin resistance (≥ 4.0) [30]. The TG:HDL-C ratio is calculated by dividing triglycerides by HDL-C levels. For lipid profiles, the cut-off values for high total cholesterol, high triglycerides, low HDL-C, and high LDL-C were 5.20, 1.70, 1.03, and 2.84 mmol/L, respectively [28]. Hypertension was categorized based on definitions of blood pressure categories used by the American Heart Association [28]: normal (< 90th percentile), elevated (≥ 90th percentile to < 95th percentile), stage 1 (≥ 95th percentile to < 95th percentile + 12 mmHg), and stage 2 (≥ 95th percentile + 12 mmHg).

### Statistical analysis

All data analyses were performed using the IBM Statistical Package for Social Sciences version 24. Baseline characteristics were determined for both the intervention and control groups. Continuous data were presented as mean and standard deviation, whereas categorical data were described in terms of frequency and percentage. Independent t-tests for continuous variables and chi-square tests for categorical variables were used to determine the differences between study groups. Analysis within group was performed using repeated measures of analysis of variance (ANOVA), whereas differences of mean changes between groups were measured using repeated measures of analysis of covariance (ANCOVA) adjusted for gender and baseline variables. A *P*-value of < 0.05 was considered statistically significant.

## Results

Table 1 shows the socio-demographic and anthropometric measures of the intervention and control groups at baseline. All socio-demographic and anthropometric measures were comparable at baseline, except for ethnicity and school location. The mean (SD) ages of adolescents were 14.20 (1.35) years in the intervention group and 14.24 (1.35) years in the control group. The majority of the adolescents in both the intervention and control groups were Malay, followed by Indian, Chinese, and other ethnic groups. Most were from urban schools in both groups.

**Table 1:** Socio-demographic and anthropometric measures of the MyBFF@school participants in secondary school

Features	Intervention (*n* = 306)	Control (*n* = 246)	*p*-value
Demographic			
Age, mean ± SD	14.20 ± 1.35	14.24 ± 1.35	0.731
Gender, n (%)			
Boys	105 (34.3)	86 (35.0)	0.928
Girls	201 (65.7)	160 (65.0)	
Ethnicity, n (%)			
Malay	242 (79.1)	186 (75.6)	0.005
Chinese	18 (5.9)	20 (8.1)	
Indian	32 (10.5)	39 (15.9)	
Others	14 (4.6)	1 (0.4)	
School location, n (%)			
Urban	175 (57.2)	178 (72.4)	< 0.001
Rural	131 (42.8)	68 (27.6)	
Anthropometric Measures			
BMI (kg/m^2^), mean ± SD	28.40 ± 4.78	28.04 ± 4.82	0.386
BMI z-score, mean ± SD	2.12 ± 0.74	2.06 ± 0.73	0.346
BMI categories, n (%)			
Overweight	142 (46.4)	132 (53.7)	0.179
Obese	123 (40.2)	90 (36.6)	
Morbidly obese	41 (13.4)	24 (9.8)	

*p*-value was calculated using independent *t*-test for continuous variables and chi-square test for categorical variables

*Abbreviations: SD* Standard deviation, *BMI* Body mass index

At baseline, the prevalence of overweight, obese and morbidly obese schoolchildren in this study population were 49.6%, 38.6% and 11.8% respectively. Morbidly obese adolescents in the intervention and control groups were 13.4% and 9.8% respectively. The prevalence of different stages of obesity among the intervention and control groups is available in Table 1.

The prevalence of pre-diabetes was 1.5%, while type 2 diabetes was 0.9%. Hypertension was detected in 20.2% of this population (16.8% with Stage 1 hypertension and 3.4% with Stage 2 hypertension). As for dyslipidemia, 8.3% had high total cholesterol, 5.8% had high triglycerides, 44.2% had high LDL-C and 54.0% had low HDL-C. The prevalence of each cardiometabolic risk among the intervention and control groups are available in Table 2.

**Table 2:** Baseline distribution of diabetes, insulin resistance, lipid profile and blood pressure status among MyBFF@school participants

Characteristics	Intervention (*n* = 306)	Control (*n* = 246)	*p*-value
**Diabetes**			
*Fasting plasma glucose* (mmol/L), mean ± SD	4.90 ± 0.75	4.81 ± 0.62	0.124
Normal (< 5.6 mmol/L), n (%)	299 (97.7)	240 (97.6)	0.933
Pre-diabetes (5.6—< 7.0 mmol/L), n (%)	4 (1.3)	4 (1.6)	
Diabetes (≥ 7.0 mmol/L), n (%)	3 (1.0)	2 (0.8)	
Insulin resistance			
*Fasting insulin* (μU/mL), mean ± SD	18.45 ± 11.33	17.21 ± 8.80	0.159
*HOMA-IR, mean ± SD	4.04 ± 2.63	3.67 ± 1.92	0.066
Insulin sensitive (< 4.0), n (%)	171 (58.4)	151 63.7)	0.212
Insulin resistance (≥ 4.0), n (%)	122 (41.6)	86 (36.3)	
Lipid profile			
*Total cholesterol* (mmol/L), mean ± SD	4.17 ± 0.69	4.10 ± 1.03	0.370
Normal (< 5.2 mmol/L), n (%)	283 (92.5)	223 (90.7)	0.444
High (≥ 5.2 mmol/L), n (%)	23 (7.5)	23 (9.3)	
*Triglycerides* (mmol/L), mean ± SD	0.98 ± 0.48	0.91 ± 0.43	0.108
Normal (< 1.7 mmol/L), n (%)	287 (93.8)	233 (94.7)	0.716
High (≥ 1.7 mmol/L), n (%)	19 (6.2)	13 (5.3)	
*HDL-C* (mmol/L), mean ± SD	1.04 ± 0.18	1.08 ± 0.29	0.037
Normal (≥ 1.03 mmol/L), n (%)	156 (51.0)	152 (61.8)	0.012
Low (< 1.03 mmol/L), n (%)	150 (49.0)	94 (38.2)	
*LDL-C* (mmol/L), mean ± SD	2.84 ± 0.71	3.10 ± 1.05	0.001
Normal (< 2.84 mmol/L), n (%)	157 (51.3)	97 (39.4)	0.006
High (≥ 2.84 mmol/L), n (%)	149 (48.7)	149 (60.6)	
*TG:HDL-C* (mmol/L), mean ± SD	0.98 ± 0.55	0.90 ± 0.49	0.072
Blood pressure			
*Systolic* (mmHg), mean ± SD	110.48 ± 12.69	111.02 ± 10.95	0.600
*Diastolic* (mmHg), mean ± SD	70.51 ± 10.28	69.73 ± 8.00	0.336
Normal (< 90th percentile), n (%)	200 (65.8)	160 (65.6)	0.159
Elevated blood pressure (≥ 90th percentile to < 95th percentile), n (%)	38 (12.5)	38 (15.6)	
Stage 1 HTN (≥ 95th percentile to < 95th percentile + 12 mmHg), n (%)	51 (16.8)	42 (17.2)	
Stage 2 HTN (≥ 95th percentile + 12 mmHg), n (%)	15 (4.9)	4 (1.6)	

*p-*value was calculated with independent* t*-test for continuous variables and chi-square test for categorical variables

^*^intervention (*n* = 304), control (*n* = 244)

*Abbreviations*: *SD* Standard deviation, *HOMA-IR* Homeostatic model assessment of insulin resistance, *HDL-C* high density lipoprotein cholesterol, *LDL-C* Low density lipoprotein cholesterol, *TG, HDL-C* Triglycerides to high density lipoprotein cholesterol ratio, *HTN* Hypertension

Table 2 shows the distribution of diabetes, insulin resistance, lipid profiles, and blood pressure status at baseline. There were no significant differences in biochemical profiles between the intervention and control groups at baseline, except for HDL-C and LDL-C which were both significantly lower in the intervention group. The prevalence rates of adolescents with pre-diabetes and diabetes in the intervention group at baseline were 1.3% and 1.0%, respectively. Meanwhile, the adolescents in the control group with pre-diabetes and diabetes accounted for 1.6% and 0.8%, respectively. Adolescents with insulin resistance accounted for 41.6% of the intervention group and 36.3% of the control group. The percentages of adolescents with elevated blood pressure (hypertension), stage 1 hypertension, and stage 2 hypertension in the intervention group were 12.5%, 16.8%, and 4.9%, respectively. The corresponding figures in the control group were 15.6%, 17.2%, and 1.6%.

BMI and cardiometabolic marker changes within and between groups after six months of intervention are presented in Table 3. At the end of the intervention, BMI z-score was significantly lower in both groups. While no significant between-group differences were observed for BMI or BMI z-score, it is important to note that BMI significantly increased (albeit small) within the intervention group compared with baseline (0.17 kg/m^2^ (95%CI (0.02, 0.32)). In addition, significant increases were also observed in triglycerides, LDL-C, fasting plasma glucose, and both systolic and diastolic blood pressure in the intervention group compared with the control group (all *p* < 0.001)*.* On the other hand*,* HDL-C showed a significant improvement in both groups post-intervention, with a greater increase in the intervention group (0.25 mmol/L (95% CI 0.23, 0.28)) than in the control group (0.18 mmol/L (95% CI 0.14, 0.21)), with significant difference detected between the two groups (*p* = 0.004). Fasting insulin showed a significant reduction in the intervention group (− 2.24 μU/mL (95% CI − 3.37, − 1.17)), whereas no significant reduction was observed in the control group (− 0.82 μU/mL (95% CI − 1.86, 0.22)). However, when comparing between groups, no significant changes were detected. Furthermore, there was a trend of reduction of HOMA-IR in the intervention group (− 0.03 (95% CI − 0.32, 0.24)) compared to the slight increase in the control group (0.05 (95% CI 0.19, − 0.29)), but no significant changes were detected between groups.

**Table 3:** Changes of BMI and cardiometabolic markers within and between groups after six months of intervention

Measures	Group	Baseline (mean ± SD)	Month-6 (mean ± SD)	Change within group, MD (95% CI)	*p*-value^a^	Change between group, MD (95% CI)	*p*-value^b^
BMI (kg/m^2^)	Intervention (*n* = 306)	28.40 ± 4.78	28.57 ± 4.94	0.17 (0.02, 0.32)	0.002	0.46 (−0.35, 0.48)	0.413
	Control (*n* = 246)	28.05 ± 4.82	28.02 ± 4.77	−0.028 (−0.19, 0.13)	0.732		
BMI z-score	Intervention (*n* = 306)	2.12 ± 0.74	2.08 ± 0.79	−0.05(−0.08, −0.02)	0.001	0.073(−0.05,0.19)	0.254
	Control (*n* = 246)	2.06 ± 0.73	1.99 ± 0.77	−0.07(−0.09, −0.05)	< 0.001		
Fasting plasma glucose (mmol/L)	Intervention (*n* = 306)	4.90 ± 0.75	5.49 ± 0.56	**0.59 (0.51, 0.67)**	** < 0.001**	**0.38 (0.28, 0.48)**	** < 0.001**
	Control (*n* = 246)	4.81 ± 0.62	5.06 ± 0.82	**0.25 (0.19, 0.31)**	** < 0.001**		
Fasting insulin (μU/mL)	Intervention (*n* = 304)	18.46 ± 11.36	16.21 ± 10.25	** − 2.24 (− 3.37, − 1.17)**	** < 0.001**	− 1.23 (− 2.62, 0.17)	0.085
	Control (*n* = 244)	17.25 ± 8.82	16.43 ± 8.56	− 0.82 (− 1.86, 0.22)	0.123		
HOMA-IR	Intervention (*n* = 304)	4.04 ± 2.63	4.01 ± 2.77	− 0.03 (− 0.32, 0.24)	0.793	− 0.70 (− 1.85, 0.45)	0.234
	Control (*n* = 244)	3.68 ± 1.93	3.73 ± 2.07	0.05 (− 0.19, 0.29)	0.676		
Total cholesterol (mmol/L)	Intervention (*n* = 306)	4.17 ± 0.69	4.76 ± 0.88	**0.59 (0.52, 0.66)**	** < 0.001**	**0.36 (0.25, 0.47)**	** < 0.001**
	Control (*n* = 246)	4.10 ± 1.03	4.34 ± 0.86	**0.23 (0.14, 0.32)**	** < 0.001**		
Triglycerides (mmol/L)	Intervention (*n* = 306)	0.98 ± 0.48	1.18 ± 0.68	**0.20 (0.13, 0.26)**	** < 0.001**	**0.26 (0.17, 0.35)**	** < 0.001**
	Control (*n* = 246)	0.91 ± 0.43	0.86 ± 0.48	** − 0.05 (− 0.09, 0.00)**	**0.060**		
HDL-C (mmol/L)	Intervention (*n* = 306)	1.04 ± 0.18	1.29 ± 0.26	**0.25 (0.23, 0.28)**	** < 0.001**	**0.06 (0.10, 0.20)**	**0.004**
	Control (*n* = 246)	1.08 ± 0.29	1.26 ± 0.25	**0.18 (0.14, 0.21)**	** < 0.001**		
LDL-C (mmol/L)	Intervention (*n* = 306)	2.84 ± 0.71	3.61 ± 0.91	**0.76 (0.70, 0.83)**	** < 0.001**	**0.30 (0.18, 0.42)**	** < 0.001**
	Control (*n* = 246)	3.10 ± 1.05	3.48 ± 0.84	**0.38 (0.26, 0.49)**	** < 0.001**		
TG:HDL-C (mmol/L)	Intervention (*n* = 306)	0.98 ± 0.55	0.99 ± 0.82	**0.01 (− 0.08, 0.09)**	**0.899**	**0.19 (0.08, 0.30)**	**0.001**
	Control (*n* = 246)	0.90 ± 0.49	0.73 ± 0.52	** − 0.16 (− 0.22, − 0.10)**	** < 0.001**		
Systolic BP (mmHg)	Intervention (*n* = 306)	110.51 ± 12.60	115.16 ± 11.61	**4.64 (3.43, 5.85)**	** < 0.001**	**3.68 (2.01, 5.35)**	** < 0.001**
	Control (*n* = 246)	111.02 ± 10.95	111.74 ± 10.96	**0.71 (−0.71, 2.14)**	**0.327**		
Diastolic BP (mmHg)	Intervention (*n* = 306)	70.52 ± 10.29	73.34 ± 8.58	**2.81 (1.69, 3.94)**	** < 0.001**	**4.29 (2.85, 5.73)**	** < 0.001**
	Control (*n* = 246)	69.73 ± 8.00	68.63 ± 8.89	** − 1.10 (− 2.37, 0.16)**	**0.088**		

Mean differences within group and between groups are given as mean values; negative changes indicate a reduction from baseline to month 6

^a^Repeated measures analysis of variance (ANOVA) adjusted for gender, significant at *p* < 0.05

^b^Analysis of covariance (ANCOVA) adjusted for gender, significant at *p* < 0.05

*Abbreviations*: *SD* Standard deviation, *MD* Mean difference, *HOMA-IR* Homeostatic model assessment of insulin resistance, *HDL-C* High-density lipoprotein cholesterol, *LDL-C* Low-density lipoprotein cholesterol, *TG:HDL-C* Triglycerides to high-density lipoprotein cholesterol ratio, *BP* Blood pressure

## Discussion

MyBFF@school was the first study to report on the cardiometabolic effect of school-based obesity intervention in Malaysia due to substantial obstacles of blood collection among schoolchildren specifically the legal and ethical issues. Worryingly, our baseline results revealed that approximately 50% of apparently healthy adolescents with obesity had high LDL-C, 40% with insulin resistance, low HDL-C, and nearly 20% had elevated blood pressure, highlighting the importance of obesity intervention. MyBFF@ school was a multi-component intervention programme which includes diet, nutritional behavioral component, physical activity, and psychological component. We hypothesized that MyBFF@school would improve cardiometabolic outcomes, specifically insulin resistance, dyslipidemia, and blood pressure, in adolescents with obesity.

Behavioral and lifestyle changes are the first line treatment for dyslipidemia in adults [31] and recommended as the cornerstone of treatment for children and adolescents [32]. However, in MyBFF@school programme improvements were only observed in the HDL-C level. Although not conclusive from this study, improvement of HDL-C is known to increase with consistent exercise [33]. The improvement of HDL-C was in parallel with other observational and intervention studies inclusive of supervised physical activity [34, 35]. Healthy lifestyle strategies, including exercise and dietary approaches, are predicted to improve the insulin resistance and related metabolic derangements in children and adolescents with obesity [36, 37]. We observed insignificant reduction of fasting insulin and HOMA-IR in the intervention group. However, we also observed increased fasting plasma glucose in both group with significant increase in the intervention group. Previous studies have shown that physical exercise have greater influence on insulin sensitivity compared to dietary modification in adolescents [38, 39]. Meta-analysis of Marson et al. [40] concluded that exercise training in general was not associated with a reduction in fasting glucose; however, reduction in fasting insulin levels and HOMA IR were observed after aerobic exercise. Having said that, research on the most effective types (aerobic, resistance, or combined) and methods (regular, exergames, or online) of exercise for enhancing insulin sensitivity is rapidly ongoing. By then, optimal exercise program can be adopted for improving insulin sensitivity in youth with obesity.

While the intervention achieved reductions in BMI z-score within both groups, the increase in BMI (albeit small) observed in the intervention group, together with rises in triglycerides, LDL-C, fasting plasma glucose, and blood pressure, highlight the complexity of metabolic responses in adolescents with obesity. These unintended findings suggest that weight-related or behavioral interventions may exert differential effects on anthropometric and metabolic outcomes. Importantly, such changes may not always align in a uniformly beneficial direction. Several explanations are possible, including physiological adaptations during puberty [41], dietary or lifestyle factors [42, 43] not fully captured in the study, or variability in adherence to intervention components [44]. The increases in cardiometabolic markers warrant further investigation, particularly in longer-term or longitudinal designs, to determine whether these represent transient fluctuations or sustained adverse effects. Future studies should therefore not only assess weight-related outcomes but also place equal emphasis on comprehensive cardiometabolic profiling to ensure interventions are both effective and safe.

Our study findings on the cardiometabolic outcomes have to be interpreted against some limitations: First, changes in obesity measures (BMI z-scores, body fatness, body weight). Several studies showed association between reduction in obesity measures due to lifestyle intervention and improvement of cardiovascular risk factors among obese children [45, 46]. However, one study showed no change in BMI and/or BMI z-score in children and adolescents with overweight or obesity seems to be related to an increase in total cholesterol and triglycerides, but not blood pressure [47]. In this study, significant change in BMI z-scores was observed which may partly affect the cardiometabolic change i.e. increase in HDL-C and reduction of fasting insulin. However, the increased in other lipid parameters and blood pressure, despite reduction (albeit small) in BMI z-score warrant further detail analysis. Secondly, MyBFF@school only measured quantitative food frequency questionnaire (FFQ) for the past one week which was adopted for FFQ on fruit and vegetable from the WHO STEPwise Approach for Surveillance of Non-Communicable Diseases [48]. Hence the intake of micro and macro nutrient cannot be taken into consideration for adjustment in our model of analysis.

MyBFF@ school protocol was intended to be easily applied and incorporated in the school settings for obesity intervention. Overall, our findings suggest that while the intervention showed promise in certain aspects, it may not be universally beneficial and requires further refinement and longer-term evaluation before it can be recommended as a safe and effective strategy for obese adolescents*.* Nevertheless, the growing prevalence of severe obesity among adolescents is of concern and for whom prevention efforts alone are unlikely to have a meaningful impact. Therefore, we suggest considering the staged treatment of pediatric obesity as suggested by the American Academy of Pediatrics [49]. Staged treatment of obesity is practice in the clinical setting to escalate obesity treatment for non-responsive individuals. Recently, Arlinghaus et al. [50] conducted a staged school-based obesity treatment model for low-income, ethnic minority youth. The program called Take Charge was an escalated obesity treatment after an establish semester-long intensive lifestyle intervention. The author reported a significant decrease in percentage of the 95th BMI percentile among those initially unresponsive to the semester-long intensive lifestyle intervention. This suggests that refining the intervention content could be beneficial for those who did not respond initially. It also illustrates that schools can play a multi-staged role in treating obesity.

## Conclusion

Although some metabolic improvements were observed in the MyBFF@school programme (e.g., reduction in fasting insulin and increase in HDL within the intervention group), several potentially unintended changes were also observed. Notably, BMI significantly increased within the intervention group, and this was accompanied by significant increases in triglycerides, LDL cholesterol, fasting plasma glucose, and blood pressure compared with controls. These findings suggest that the intervention may have had mixed metabolic effects, and the observed weight gain could partly explain the unintended changes. Therefore, our results highlight the need for cautious interpretation and underscore the importance of considering both beneficial and detrimental outcomes when evaluating intervention effects.

## Data Availability

All relevant data are within the paper.

## Abbreviations

ANCOVA: Analysis of covariance
ANOVA: Analysis of variance
BMI: Body mass index
HDL-C: High-density lipoprotein cholesterol
HOMA-IR: Homeostatic model assessment of insulin resistance
LDL-C: Low-density lipoprotein cholesterol
MyBFF@school: My Body is Fit and Fabulous at School
SD: Standard deviation
T2DM: Type 2 diabetes mellitus
TG: HDL-C ratio: Triglycerides to high-density lipoprotein cholesterol ratio
WHO: World Health Organization
